# The Safety of Early Discharge Following Transcatheter Aortic Valve Implantation Among Patients in Northern Ontario and Rural Areas Utilizing the Vancouver 3M TAVI Study Clinical Pathway

**DOI:** 10.1016/j.cjco.2022.08.005

**Published:** 2022-08-13

**Authors:** George Hanna, Derek Macdonald, Bindu Bittira, Eric Horlick, Noman Ali, Rony Atoui, Abdulrahman Alqahtani, Neil Fam, Mohammed Shurrab, Joanne Spadafore, Julie Allen, Asim Cheema, Bhanu Nalla, Carly Pulkkinen, Sylvain Cote, Hooman Hennessey, Melissa Stringer, Suzanne Leblanc, Joanne Collin, John Fenton, Mathieu Rheault-Henry, Sandra Lauck, Janarthanan Sathananthan, David Wood, Sami Alnasser

**Affiliations:** a Department of Cardiology, St Michael’s Hospital, University of Toronto, Toronto, Ontario, Canada; b Division of Cardiac Surgery, Health Sciences North, Sudbury, Ontario, Canada; c Northern Ontario School of Medicine, Sudbury, Ontario, Canada; d Division of Cardiology, Peter Munk Cardiac Centre, Toronto General Hospital, Toronto, Ontario, Canada; e Division of Cardiology, Health Sciences North, Sudbury, Ontario, Canada; f Southlake Regional Health Center, Newmarket, Ontario, Canada; g Division of Anaesthesia, Health Sciences North, Sudbury, Ontario, Canada; h Division of Radiology, Health Sciences North, Sudbury, Ontario, Canada; i Division of Vascular Surgery, Health Sciences North, Sudbury, Ontario, Canada; j University of British Colombia Innovation Centre, Vancouver, British Colombia, Canada

## Abstract

**Background:**

Early hospital ( < 48 hours) discharge following transcatheter aortic valve implantation (TAVI) is an increasingly adopted practice; however, data on the safety of such an approach among patients residing in North Ontario, including remote and medically underserved areas, are lacking.

**Methods:**

This retrospective study included patients who underwent TAVI in Sudbury, Ontario. The safety of early discharge after implementation of the Vancouver 3M (multidisciplinary, multimodality, but minimalist) clinical pathway was assessed. The primary endpoint was 30-day mortality. Resource utilization before vs after 3M clinical pathway implementation was also compared.

**Results:**

A total of 291 patients who underwent TAVI between 2012 and 2021 were included in the study. One in-hospital death (0.6%) occurred after the 3M clinical pathway implementation, with no mortality observed beyond hospital discharge. Eleven patients (6.7%) required rehospitalization within 30 days. The need for mechanical ventilation and surgical vascular cut-down declined from 100% and 97%, respectively, at baseline, to 6% and 2%. The number of patients receiving TAVI on a given procedural day increased from 2 to 3 patients. The median post-TAVI hospital length of stay decreased from 5 days (2-6 days) to 1 day (1-3 days) after 3M clinical pathway implementation.

**Conclusions:**

Following TAVI, early discharge of selected patients residing in Northern Ontario, including rural areas, using the Vancouver 3M clinical pathway was associated with favourable outcomes, short length of stay, and more-efficient resource utilization. These data can help improve healthcare efficiency and bridge variations in TAVI funding and accessibility in underserved locations.

## Graphical abstract

**Figure:**
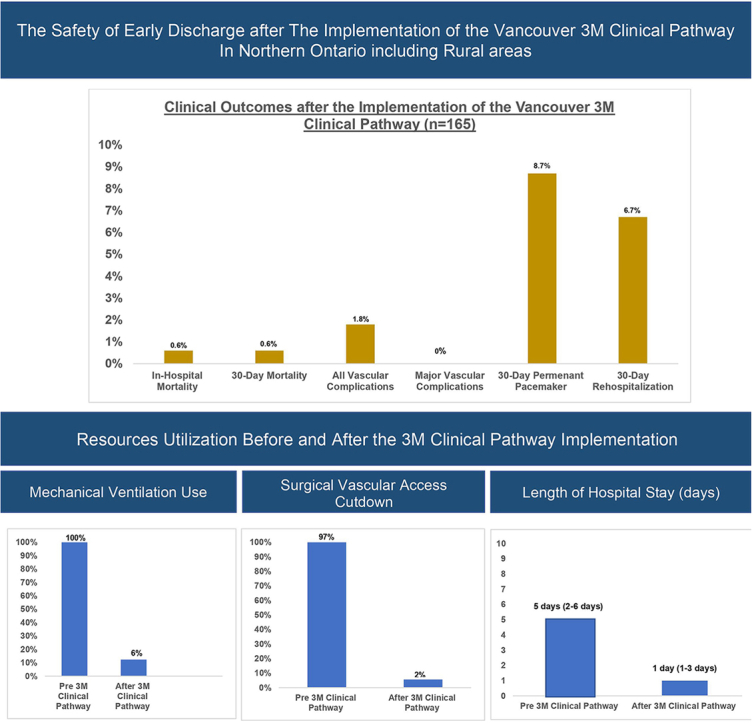

## Résumé

**Contexte:**

Il est de plus en plus admis d’accorder un congé rapide de l’hôpital (< 48 heures) après une implantation valvulaire aortique par cathéter (IVAC); toutefois, on ne dispose pas de données sur l’innocuité de cette pratique pour les patients du nord de l’Ontario, y compris ceux qui résident en régions éloignées moins bien desservies par les services médicaux.

**Méthodologie:**

Cette étude rétrospective a porté sur des patients ayant subi une IVAC à Sudbury (Ontario). L’innocuité d’un congé rapide après l’implantation selon le parcours de soins Vancouver 3M (multidisciplinaire, multimodal, mais minimaliste) a été évaluée. Le principal paramètre d’évaluation était la mortalité à 30 jours. Une comparaison de l’utilisation des ressources avant et après la mise en œuvre du parcours de soins 3M a également été effectuée.

**Résultats:**

Au total, 291 patients ayant subi une IVAC entre 2012 et 2021 ont été inclus dans l’étude. Un décès à l’hôpital (0,6 %) est survenu après la mise en œuvre du parcours de soins 3M, et aucune mortalité n’a été relevée après le congé de l’hôpital. Onze patients (6,7 %) ont dû être réhospitalisés dans les 30 jours suivants. Le recours à la ventilation mécanique et à la dénudation vasculaire a chuté, passant de 100 % et 97 % au départ, respectivement, à 6 % et 2 %. Le nombre de patients par jour d’intervention subissant une IVAC est passé de deux à trois patients. À la suite de la mise en œuvre du parcours de soins 3M, la durée médiane du séjour à l’hôpital après une IVAC est passée de cinq jours (deux à six jours) à un jour (un à trois jours).

**Conclusions:**

Après une IVAC, le congé rapide de patients sélectionnés habitant dans le nord de l’Ontario, y compris ceux habitant en région rurale, selon le parcours de soins Vancouver 3M a été associé à des résultats de santé favorables, à une durée courte d’hospitalisation et à une utilisation plus efficace des ressources. Ces données peuvent contribuer à améliorer l’efficacité des soins de santé et à combler des écarts liés aux variations du financement et de l’accessibilité des IVAC dans les régions moins bien desservies.

Transcatheter aortic valve implantation (TAVI) has become an established treatment for patients with severe aortic stenosis.^1^^,^^2^ The advancement in technology and improved operator experience has enabled the adoption of minimally invasive measures during TAVI, eliminating the routine need for general anaesthesia and surgical vascular access cut-down. These minimally invasive measures have streamlined the procedure and enabled early discharge of patients following TAVI. The Vancouver 3M (multidisciplinary, multimodality, but minimalist) TAVI study demonstrated favourable outcomes with early post-TAVI discharge among selected patients.^3^ In order to achieve early discharge safely, the 3M protocol has been published to facilitate early ambulation and a return to baseline mobility prior to patient discharge.^4^ Furthermore, early hospital discharge post-TAVI improves resources utilization, which has been particularly important during the COVID-19 pandemic and similar situations.

Although the minimalist approach to TAVI and the associated early discharge is an increasingly adopted practice, the translation of this approach can be challenging among patients residing in rural areas. Health Sciences North (HSN) in Sudbury, Ontario serves a large rural area across Northeast Ontario. Patients from remote areas travel significant distances to receive TAVI at more-urban centers. Early discharge for patients residing in remote or medically underserved parts of Northern Ontario has been a concern; limited diagnostic and therapeutic capabilities in some of these regions can pose difficulties with identification and treatment of TAVI-related complications, which may manifest several days after discharge. Furthermore, if a complication is encountered, transfer of patients back to the implanting center can be hindered in Northern Ontario by logistic issues and extreme weather conditions. As a result, the safety of early discharge for such patient populations is unknown.

The aim of the present study is to examine the safety of early discharge after TAVI among patients in Northern Ontario, and rural areas, utilizing the Vancouver 3M TAVI study clinical pathway.

## Materials and Methods

**Figure 1:**
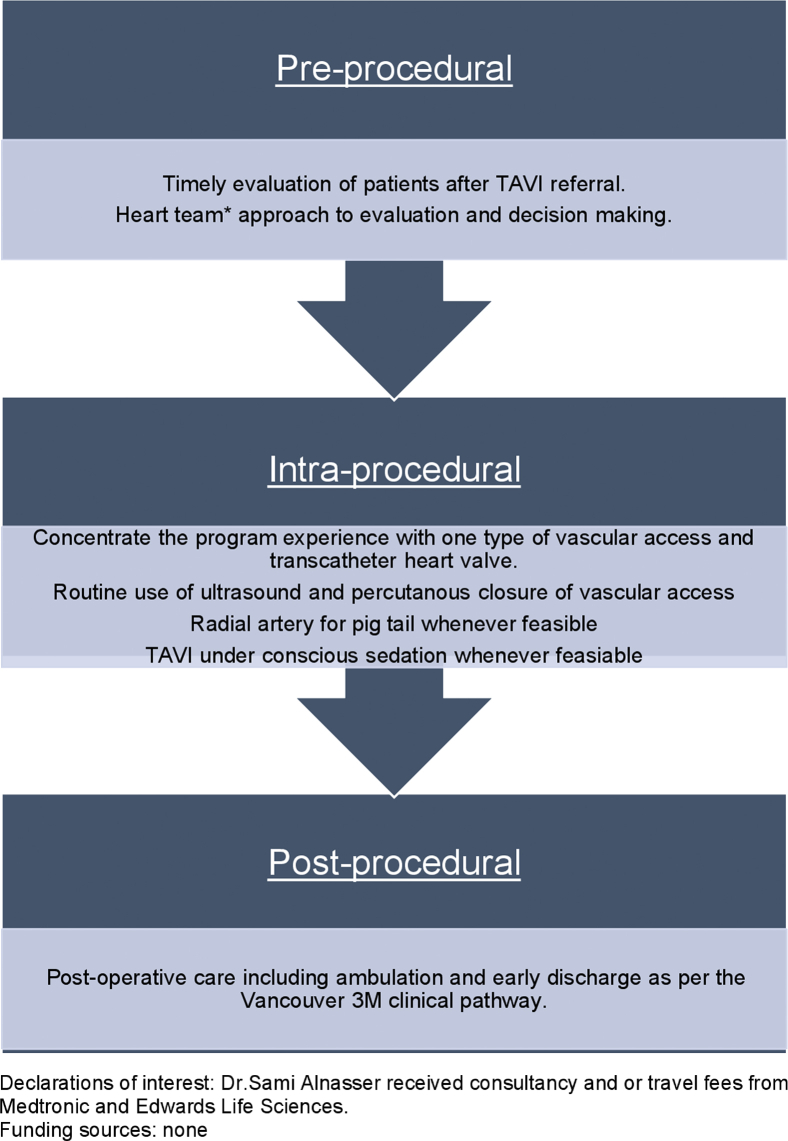
Updated institutional transcatheter aortic valve implantation (TAVI) workflow and practices. ∗Multidisciplinary heart team that included cardiac nurses, cardiologists, cardiac surgery, vascular surgery, and anaesthesiology. Vancouver 3M, multidisciplinary, multimodality, but minimalist.

This retrospective cohort study was conducted at HSN in Sudbury, Ontario. Patients who underwent TAVI between 2012 and 2021 were included. Institutional TAVI practices at HSN were reviewed in 2017 by a multidisciplinary “heart team” that included cardiac nurses, cardiologists, cardiovascular surgeons, and anaesthesia specialists. This approach resulted in the implementation of the Vancouver 3M TAVI clinical pathway at HSN in March 2018, including transitioning to procedural conscious sedation and percutaneous vascular access closure (Fig. 1).

When feasible, the team aimed to discharge patients with heart failure, after management and completion of the TAVI workup tests, in order to expedite outpatient TAVI to minimize the acute kidney injury risk and allow for improved patient ambulation.

The primary objective of this study was to assess the safety of early discharge after adoption of the Vancouver 3M study clinical pathway. A secondary analysis was conducted to compare the resource utilization before vs after the pathway implementation. The primary endpoint was 30-day mortality. Secondary endpoints included 30-day rehospitalizations, need for a permanent pacemaker, and the hospital length of stay (LOS). Other outcomes include bleeding or vascular access complications as described by the Valve Academic and Research Consortium 3 definitions (Supplemental Tables S1 and S2).^5^ TAVI volume was defined according to the number of cases performed annually. Utilizing the 3M study definition, a low-volume center was defined as one performing less than 100 TAVI procedures per year.^3^

### Procedural and postoperative care

Percutaneous vascular access was obtained using a 4-Fr micro-puncture needle and a catheter under ultrasound guidance. The radial artery was preferentially used as a secondary access site for positioning of a pigtail catheter at the aortic root. An additional arterial line was used for invasive hemodynamic monitoring. Deployment of the transcatheter heart valve (THV) was done under rapid pacing, which was achieved using a 5-Fr balloon-tipped transvenous pacemaker (TVPM) wire positioned within the right ventricle. Implantations were assessed using aortic root angiography as well as transthoracic echocardiography. Patients were anticoagulated with heparin, aiming for an activated clotting time of between 250 and 350 seconds. Reversal of anticoagulation with protamine was undertaken only in selected cases. The TVPM was removed after balloon-expandable THV implantations, except in cases with major preexisting or newly developed conduction abnormalities. The TVPM was routinely left for 24 hours following self-expanding THV implantations, except after treatment of degenerated surgical bioprostheses (valve-in-valve).

The Vancouver 3M clinical pathway to encourage early ambulation and removal of catheters was adopted (Supplemental Table S3), and discharge within 24 hours was encouraged in cases in which the 3M early-discharge criteria (Supplemental Table S4) were met and patients were deemed safe for discharge from an ambulation point of view, per local protocol.^4^ Patients with new persistent left bundle branch block were discharged home on the following day, except in cases of progressive conduction abnormalities, baseline right bundle branch block, or if occurred after self-expanding valve implantations. For patients with new left bundle branch block, a 12-lead electrocardiogram was recommended within 3 days after discharge, and an outpatient Holter monitor was organized during follow-up. A single antiplatelet agent was used for postprocedural thromboprophylaxis, unless standard indications for anticoagulation were present.

### Statistical analysis

Continuous variables are presented as a mean and standard deviation, or as a median and interquartile range, and categorical variables are presented as an absolute number and percentage.

## Results

A total of 291 patients who underwent TAVI between 2012 and 2021 at HSN were included in this study. This number comprised 126 and 165 patients, respectively, who had their TAVI before vs after adoption of the Vancouver 3M clinical pathway. The mean age for the overall cohort was 81 years (+/-7 years), and patients predominantly (99%) underwent TAVI via the transfemoral approach. Other baseline characteristics are shown in Table 1. The annual HSN TAVI volume is shown in Supplemental Figure S1.

**Table 1:** Baseline characteristics of the cohorts before vs after implementation of the Vancouver 3M (multidisciplinary, multimodality, but minimalist) clinical pathway

Baseline characteristics	Pre-implementation (n = 126)	Post- implementation (n = 165)
Age, y	81 (± 7)	81 (± 8)
Female	48 (38)	75 (45)
Coronary artery disease	57 (45)	70 (42)
Hypertension	114 (90)	160 (96)
Diabetes	25 (20)	52 (32)
Atrial fibrillation	33 (26)	49 (30)
PPM implantation	12 (10)	16 (10)
History of CKD	21 (17)	47 (28)
CKD requiring RRT	3 (2)	7 (4)
History of prior stroke	12 (10)	15 (9)
COPD	8 (6)	25 (15)
History of liver cirrhosis	3 (2)	9 (5)
LVEF < 55%	20 (16)	28 (17)
AVA, cm^2^	0.7 (± 0.2)	0.7 (± 0.18)
Mean aortic valve gradient, mm Hg	40 (± 14)	45 (± 12)
Balloon expandable system	42 (33)	143 (87)
Self-expanding system	85 (67)	22 (13)
Mechanical hemodynamic support utilization∗	0	2 (1.2)

Values are mean (± standard deviation), or n (%).

AVA, aortic valve area; CKD, chronic kidney disease; COPD: chronic obstructive pulmonary disease; IQR, 25th and 75th interquartile ranges; LVEF, left ventricle ejection fraction; PPM: permanent pacemaker; RRT, renal replacement therapy.

∗ Intra-aortic balloon pump in one patient and extracorporeal membrane oxygenation support in another.

After the 3M TAVI clinical pathway was adopted, one in-hospital death occurred (0.6%) due to intractable ventricular arrhythmia and progressive cardiogenic shock despite a successful THV implantation. No additional mortality was observed within 30 days after hospital discharge. One patient (0.6%) had a transient ischemic attack, with no residual neurologic deficits. One major non-access-related Valve Academic and Research Consortium 3 bleeding event occurred (0.6%). Three minor and no major vascular complications occurred. The 30-day rate of permanent pacemaker implantations was 8.7%. THV was successfully implanted in all but one patient, in whom TAVI was aborted due to concerns of coronary obstruction, but who later underwent successful self-expanding-THV implantation.

The median post-TAVI LOS was 1 day (interquartile range: 1-3). Two patients who required mechanical hemodynamic support required 1 day of monitoring in the critical care unit and were both discharged home after 48 hours. In follow-up, 11 patients (6.7%) required rehospitalization within 30 days. Rehospitalization was due to cardiac causes in 3 patients (1 due to chest pain and 2 due to heart failure exacerbation), whereas the remaining causes were noncardiac, including acute kidney injury (1), anemia (1), fall (1), infection (3), and nonspecific abdominal pain (2). Other outcomes, including those occurring prior to the implementation of the 3M clinical pathway, are shown in Table 2.

**Table 2:** Outcomes of the cohorts before vs after implementation of the Vancouver 3M (multidisciplinary, multimodality, but minimalist) clinical pathway

Outcome	Pre-implementation (n = 126)	Post-implementation (n = 165)
30-day mortality	7 (5.6)	1 (0.6)
30-day rehospitalizations	36/119 (30)	11/164 (6.7)
In-hospital mortality	7 (5.5)	1 (0.6)
Requirement of second THV implantation	4 (3.2)	0 (0)
All vascular complications	14 (11)	3 (1.8)
Major vascular complications	3 (2.4)	0 (0)
PPM implantation	14/107 (13)	13/148 (8.8)
Post TAVI length of stay, d, median (IQR)	5 (2–6)	1 (1–3)

Values are n (%), unless otherwise specified.

IQR, interquartile range; PPM, permanent pacemaker; TAVI, transcatheter aortic valve implantation; THV, transcatheter heart valve.

**Figure 2:**
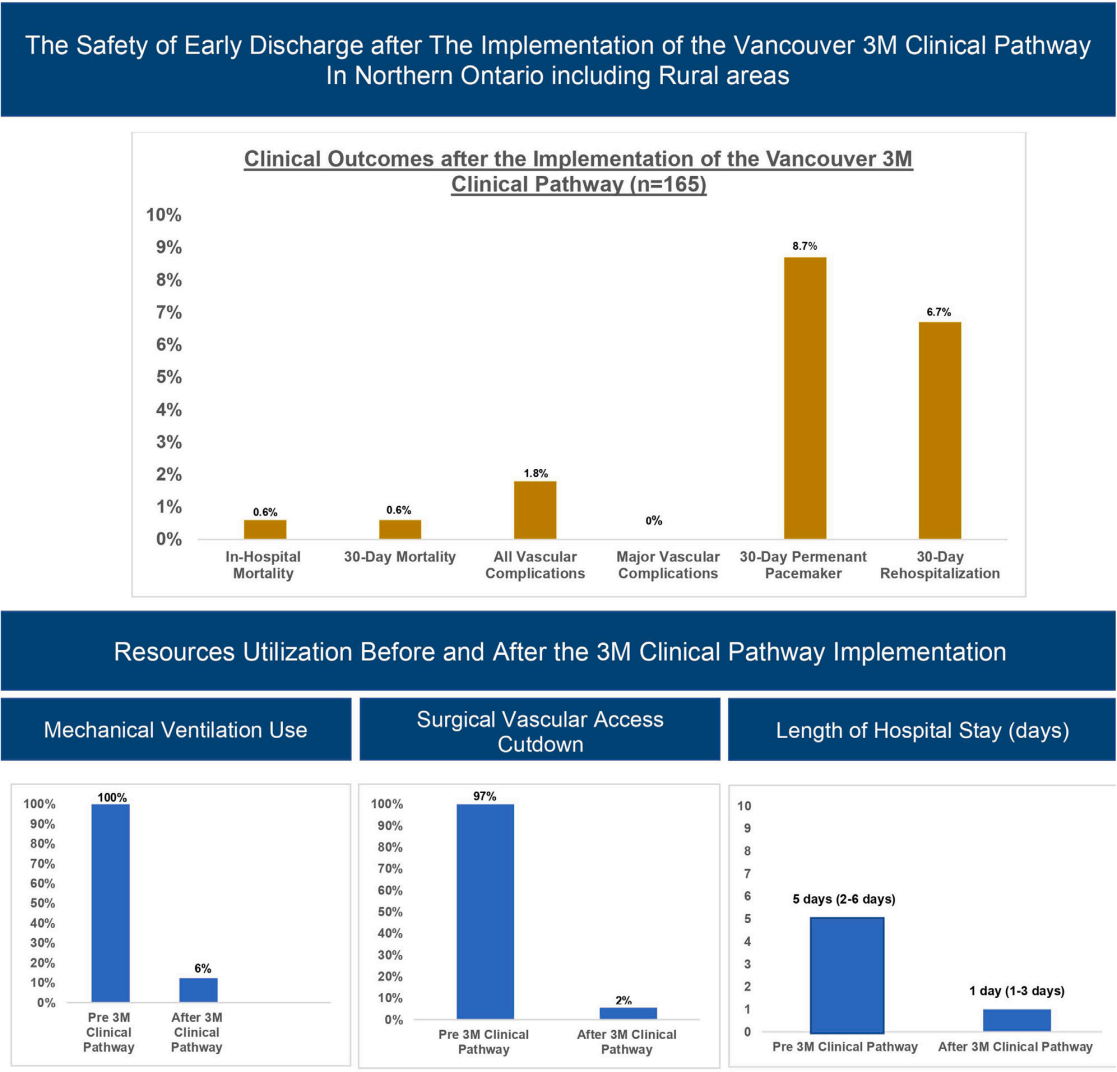
Clinical outcomes and resource utilization after the implementation of the Vancouver 3M (multidisciplinary, multimodality, but minimalist) clinical pathway.

The need for procedural mechanical ventilation and surgical vascular access cut-down declined from 100% and 97% at baseline, to 6% and 2%, respectively after adoption of the 3M clinical pathway (Fig. 2). Similarly, the number of patients receiving TAVI on a given procedural day increased from 2 to 3, and the median LOS declined from 5 days (2-6 days) to 1 day (1-3 days).

## Discussion

This analysis is the first, to our knowledge, to examine the outcomes of patients undergoing TAVI in Northern Ontario, and it yields several important findings. First, it demonstrates the feasibility of transitioning to a minimalist TAVI approach in a lower-volume center by utilization of a multidisciplinary approach, interprofessional planning, and institutional mentorship. Second, the use of the 3M clinical pathway enabled safe early discharge after TAVI for patients residing in Northern Ontario, including rural areas, with short hospital stay and improved resource utilization. Third, our analysis validates the use of the Vancouver 3M clinical pathway among selected patients from rural areas in Northern Ontario, and similar populations.

Canadian TAVI penetration is low in comparison to its European and American counterparts,^6^^,^^7^ which results in longer waiting lists for patients.^8^^,^^9^ In fact, the waiting time to TAVI for patients in Ontario has historically exceeded that for surgical aortic valve replacement. Patients on the waiting list for TAVI are at risk of unplanned hospitalization or death prior to receiving treatment.^8^^,^^9^ As of 2019, 28 Canadian sites were undertaking TAVI, the majority being tertiary centre hospitals in large, metropolitan cities where subspecialty care is more readily accessible. Despite the rise in the total number of TAVI procedures in Canada, the increase is not meeting the significant growth in demand due to the aging population and the expansion of TAVI indications to lower-risk populations.^6^ This problem is further compounded for patients that reside in rural areas, due to the inequity in access to TAVI that exists within Canada.^6^ Northern Ontario is an example of this, where issues exist pertaining to resource availability, program capacity, and limited TAVI funding allocation compared to centres in southern Ontario. As a result, one of the key priorities for Canadian TAVI programs must be to increase efficiency.

Based on our experience, streamlining the TAVI process (Fig. 1) and adopting minimally invasive measures were keys to improving resource utilization. After adoption of these measures, for instance, the need for procedural mechanical ventilation was significantly reduced (Fig. 2), which is critically important, particularly during periods of strain on the healthcare system, such as the COVID-19 pandemic. In addition, a welcomed result of implementation of changes to facilitate early discharge was an improvement in program capacity, enabling the treatment of more patients in a given procedural day.

Although early discharge post-TAVI is desirable, several valid concerns accompany such an approach for centres that serve rural catchment areas. First, despite significant advances in efficacy and safety, TAVI is still associated with potentially fatal complications that may manifest only several days after the procedure. Second, Canadians living in rural and lower-density populations tend to be older and less affluent, and have a greater prevalence of comorbidities that may increase the risk of rehospitalization.^10^ Third, patients living in rural areas have limited access to care, as they are served by only 8% of the physicians practicing in Canada with limited diagnostic and therapeutic capabilities.^11^ Moreover, re-transfer back to the implantation centre can be hindered by logistic reasons and weather conditions. These factors represent real barriers to early discharge following TAVI in North Ontario. The Vancouver 3M study suggested that early discharge after minimally invasive TAVI is safe, with low 30-day mortality and short LOS. However, the 3M study participating centres enrolled patients from metropolitan cities with limited representation of rural populations analogous to those in Northern Ontario. The present study demonstrated that adoptation of the 3M clinical pathway can lead to effective translation of the study outcomes to a geographically rural area in Canada. We observed no 30-day mortality beyond hospital discharge, a short hospital stay, and a low rehospitalization rate.

In order to transition to a protocol facilitating early discharge, robust discharge policy and follow-up arrangements were critical. Patients were provided with clear and direct contact information, including access to the implantation team after their discharge. This approach, in our experience, alleviated patient and family anxiety, and helped to minimize rehospitalization.

The COVID-19 pandemic has placed a premium upon maximizing virtual care. For instance, early virtual follow-up has been shown to facilitate safe early discharge for patients following ST segment elevation myocardial infarction (STEMI).^12^ In addition to virtual or telephone visits, developing technologies such as remote cardiac monitoring with semi-live transmission may further support safe early discharge post-TAVI by allowing early recognition of patients at risk of progressive conduction abnormalities.

Although promising, our study findings need to be interpreted with the acknowledgement of several limitations. Our data represent a single-centre experience with a relatively small sample size, and so the generalizability of our findings may be limited. The study is also retrospective, making the data dependent on available clinical reports. In addition, the cohort pre-3M protocol implementation included early TAVI experience for the centre, which may negatively impact clinical and procedural outcomes for this cohort. Also, patients in our study cohort were predominantly treated with balloon-expandable THV platforms, and so results may not be applicable to patients treated with other THVs, for whom delayed conduction abnormalities may be more frequent. Moreover, our findings should not be extrapolated to hospitals that do not meet the established operator and institution recommendations and requirements, including the availability of services such as cardiovascular surgery and interventional radiology.^7^^,^^13^ Last, data on the impact of this initiative on the number of patients needing to be referred to urban centres for TAVI after the implementation of the 3M pathway are not available to us and therefore are not reported here. Such data can be a subject for future analysis.

## Conclusion

As early discharge after TAVI becomes the standard in larger metropolitan cities, our study shows that early discharge post-TAVI utilizing the Vancouver 3M clinical pathway is applicable to a lower-volume centre caring for rural catchment areas in Northern Ontario and is associated with safe outcomes, a short hospital stay, and more-efficient utilization of hospital resources. These data may support improved funding allocation to centres in Northern Ontario and rural hospitals, which may bridge intra-provincial variation in TAVI utilization, establish accessibility of TAVI to patients in underserved locations, and reduce TAVI wait times.
