# Supplementary material for: The Safety of Early Discharge Following Transcatheter Aortic Valve Implantation Among Patients in Northern Ontario and Rural Areas Utilizing the Vancouver 3M TAVI Study Clinical Pathway
Source: CJC Open. 2022 Aug 13;4(12):1053–9. doi: 10.1016/j.cjco.2022.08.005 (PMC9764127; doi:10.1016/j.cjco.2022.08.005)

SUPPLEMENTARY MATERIAL

Supplemental Table S1. VARC-3 Bleeding complications definitions.

Supplemental Table S2. VARC-3 Vascular complications definitions.

Supplemental Table S3. The Vancouver 3M post TAVI Clinical Pathway.

Supplemental Table S4. The Vancouver 3M Early Discharge Criteria.

Supplemental Figure S1. Health Science North TAVI volume by fiscal year.

Supplemental Table S1. VARC-3 bleeding complications definitions.

**Type 1**	Overt bleeding that does not require surgical or percutaneous intervention,<br>but does require medical intervention by a health care professional,<br>leading to hospitalization, an increased level of care, or medical evaluation<br>(BARC 2)<br>n Overt bleeding that requires a transfusion of 1 unit of whole blood/red<br>blood cells (BARC 3a)
**Type 2**	Overt bleeding that requires a transfusion of 2–4 units of whole blood/<br>red blood cells (BARC 3a)<br>n Overt bleeding associated with a hemoglobin drop of >3 g/dL<br>(>1.86 mmol/L) but <5 g/d (<3.1 mmol/L) (BARC 3a)
**Type 3**	Overt bleeding in a critical organ, such as intracranial, intraspinal,<br>intraocular, pericardial (associated with hemodynamic compromise/<br>tamponade and necessitating intervention), or intramuscular with<br>compartment syndrome (BARC 3b, BARC 3c)<br>* Overt bleeding causing hypovolemic shock or severe hypotension<br>(systolic blood pressure <90 mmHg lasting >30 min and not responding<br>to volume resuscitation) or requiring vasopressors or surgery (BARC 3b)<br>n Overt bleeding requiring reoperation, surgical exploration, or reintervention<br>for the purpose of controlling bleeding (BARC 3b, BARC 4)<br>* Post-thoracotomy chest tube output ≥ 2 L within a 24-h period (BARC 4)<br>* Overt bleeding requiring a transfusion of ≥ 5 units of whole blood/red blood cells (BARC 3a)<br>Overt bleeding associated with a hemoglobin drop 5 g/dL (≥3.1 mmol/L) (BARC 3b).
**Type 4**	Overt bleeding leading to death. Should be classified as:<br>* Probable: Clinical suspicion (BARC 5a)<br>* Definite: Confirmed by autopsy or imaging (BARC 5b)

Supplemental Table S2. VARC-3 vascular complications definitions.

**Vascular complications**	
**Major**	One of the following:<br>• Aortic dissection or aortic rupture<br>• Vascular (arterial or venous) injury (perforation, rupture, dissection, stenosis, ischemia, arterial or venous thrombosis including pulmonary embolism, arteriovenous fistula, pseudoaneurysm, hematoma, retroperitoneal hematoma, infection) or compartment syndrome resulting in death, VARC type ≥2 bleeding, limb or visceral ischemia, or irreversible neurologic impairment<br>• Distal embolization (non-cerebral) from a vascular source resulting in death, amputation, limb or visceral ischemia, or irreversible end-organ damage<br>• Unplanned endovascular or surgical intervention resulting in death, VARC type ≥ 2 bleeding, limb or visceral ischemia, or irreversible neurologic impairment<br>• Closure device failure resulting in death, VARC type ≥2 bleeding, limb or visceral ischemia, or irreversible neurologic impairment
**Minor**	One of the following:<br>• Vascular (arterial or venous) injury (perforation, rupture, dissection, stenosis, ischemia, arterial or venous thrombosis including pulmonary embolism, arteriovenous fistula, pseudoaneurysm, hematoma, retroperitoneal hematoma, infection) not resulting in death, VARC type ≥ 2 bleeding, limb or visceral ischemia, or irreversible neurologic impairment<br>• Distal embolization treated with embolectomy and/or thrombectomy, not resulting in death, amputation, limb or visceral ischemia, or irreversible end organ damage.<br>• Any unplanned endovascular or surgical intervention, ultra-sound guided compression, or thrombin injection, not resulting in death, VARC type ≥ 2 bleeding, limb or visceral ischemia, or irreversible neurologic impairment<br>• Closure device failure not resulting in death, VARC type ≥ 2 bleeding, limb or visceral ischemia, or irreversible neurologic impairment
**Access related nonvascular complications**	
**Major**	One of the following:<br>• Non-vascular structure, non-cardiac structure perforation, injury, or infection resulting in death, VARC type ≥ 2 bleeding, irreversible nerve injury or requiring unplanned surgery or percutaneous intervention<br>• non-vascular access site (e.g. trans-apical left ventricular) perforation, injury, or infection resulting in death, VARC type ≥2 bleeding, irreversible nerve injury or requiring unplanned surgery or percutaneous intervention
**Minor**	One of the following:<br>• non-vascular structure, non-cardiac structure perforation, injury, or infection not resulting in death, VARC type ≥ 2, irreversible nerve injury, or requiring unplanned surgery or percutaneous intervention<br>• non-vascular access site (e.g. trans-apical left ventricular) perforation, injury, or infection not resulting in death, VARC type $2 bleeding, irreversible nerve injury or requiring unplanned surgery or percutaneous intervention

Supplemental Table S3. The Vancouver 3M post TAVI Clinical Pathway.

	0-6H	6-12H	12-18H	18-24H	24-36H
Vital signs	Q15 minÅ~4<br>Q1 hÅ~3	Q4 h			
Cardiac rhythm	Continuous			May discontinue for intermittent self-care	
Vascular access	Q15 minÅ~4<br>Q1 hÅ~3	Q4			Q8
Neuro vital signs and Cincinnati Stroke Scale assessment	Q15 minÅ~4<br>Q30 minÅ~2<br>Q1 hÅ~3	Q4 h			
Pain and discomfort	Assess and treat access site and back/postural pain/ discomfort as required		No pain/discomfort anticipated		
Laboratory work and tests	12-lead ECG<br>eGFR and CBC	If local anesthesia procedure and TTE not done at<br>end of procedure: TTE (bedside in unit if possible)		12-lead ECG<br>eGFR and CBC	
Invasive monitoring equipment	Avoid urinary catheter				
	Monitor central venous and peripheral arterial catheters<br>as per standard protocols	Remove central venous catheter<br>Remove peripheral arterial line	Maintain peripheral intravenous saline lock		Remove peripheral intravenous saline lock<br>before discharge home
Mobilization and activity	Bed rest<br>Head of bed FlatÅ~2 h<br>Then ↑ at 30Åã	Dangle to standing position at bedside<br>Transfer to commode<br>Mobilize short distance<br>in room	Transfer to commode<br>Up in chair for meals<br>Mobilize short distance in room<br>Encourage self-care behavior<br>Mobilize short distance outside of room<br>Facilitate uninterrupted rest/sleep and return to diurnal cycle		Up in chair for meals<br>Mobilize for 5–10 min<br>every 4–6 h<br>Encourage self-care<br>behavior<br>Facilitate rest
Elimination	Assess need for elimination	Mobilize to commode or<br>to standing position	Mobilize to commode<br>or washroom	Mobilize to washroom with assistance	
Hydration	NPO until hemostasis and confirmed clinical stability<br>IV 50–75 cc/h	If LVEF ≥50%: encourage fluids<br>If LVEF <50%: encourage fluids within limit of preprocedure fluid restrictions			
Nutrition		Light dinner up in chair	Up in chair for all meals<br>Encourage nutritional intake and preferred foods<br>Goal: 3 meals and 1–2 snacks/24 h		
Communication		Communicate early with the multidisciplinary team any clinical variables that may affect goals of care and<br>to identify opportunities to maintain patient on clinical pathway			
Patient teaching	Provide patient teaching<br>about maintaining vascular hemostasis	Provide coaching to support the facilitated reconditioning interventions (eg,<br>motivation for mobilization)<br>Begin discharge teaching			Complete discharge teaching<br>Provide vascular access<br>minor ooze dressing kit
Discharge planning		Confirm discharge plan<br>with patient and family		Assess readiness for<br>discharge	Confirm discharge criteria

Supplemental Table S4. The Vancouver 3M Early Discharge Criteria.

**Monitoring**
Completion and review of post-procedure transthoracic echocardiogram to confirm acceptable bioprosthetic hemodynamics with the absence of delayed complications
Absence of persistent intraventricular conduction delay
Absence of vascular access site complications
Absence of laboratory contraindications. If Hgb <100 g/L and eGFR <30 mL/min, obtain and review outpatient bloodwork 2 and 4 d after discharge
Facilitated reconditioning
Return to baseline mobilization
Absence of elimination issues (eg, urinary retention)
**Communication**
Multidisciplinary agreement of safety of discharge
Confirmation of discharge plan with patient/family
Confirmation of availability of social support during the initial 48 h after discharge
Completion of verbal discharge teaching and confirmation of patients/
family's understanding of the discharge guidelines and provision of written
discharge education resources and prescription of medications

eGFR indicates estimated glomerular filtration rate; and Hgb, hemoglobin

Supplemental Figure S1. Health Sciences North TAVI volume by fiscal year.

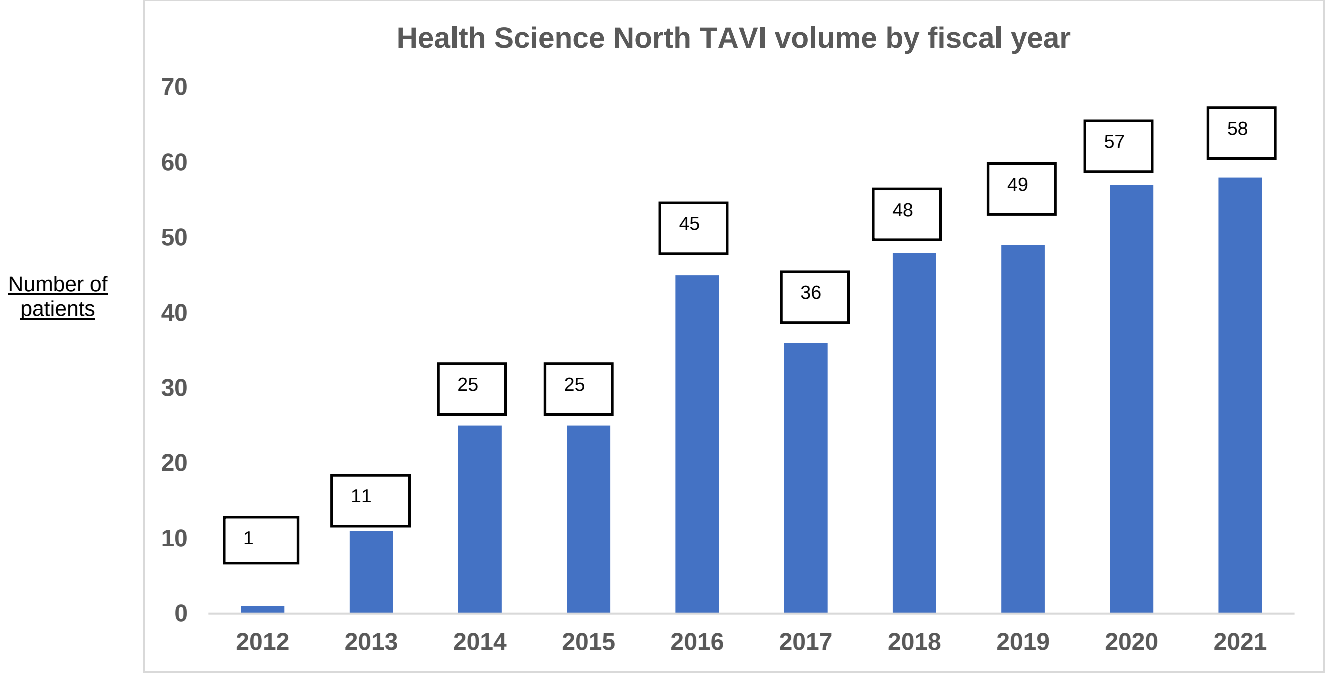

Supplement: Supplementary Material [file mmc1.pdf]
